# Anti-SARS-CoV-2 Antibody Level Is Associated with a History of COVID-19 Infection and mRNA Vaccination in Patients with Diabetes

**DOI:** 10.3390/vaccines11091424

**Published:** 2023-08-27

**Authors:** Is Asma’ul Haq Hataul, Nanny Natalia M. Soetedjo, Josephine Debora, Marita Restie Tiara, Hofiya Djauhari, Evan Susandi, Bachti Alisjahbana, Rudi Wisaksana, Hikmat Permana

**Affiliations:** 1 Internal Medicine Department, Hasan Sadikin General Hospital, Faculty of Medicine, Universitas Padjadjaran, Bandung 40161, Indonesia; asmaul18001@mail.unpad.ac.id (I.A.H.H.);; 2 Faculty of Medicine, Universitas Pattimura, Ambon 97233, Indonesia; 3 Division of Endocrinology and Metabolism, Internal Medicine Department, Hasan Sadikin General Hospital, Faculty of Medicine, Universitas Padjadjaran, Bandung 40161, Indonesia; 4 Research Center for Care and Control of Infectious Diseases (RC3ID), Universitas Padjadjaran, Bandung 40161, Indonesia

**Keywords:** COVID-19, diabetes, humoral immunity, vaccines, antibody

## Abstract

Type 2 diabetes mellitus (T2DM) is associated with higher severity and mortality in SARS-CoV-2 infections. Vaccination has been encouraged to boost immunity and prevent these unfortunate outcomes. Few studies have evaluated antibody levels after COVID-19 vaccination in patients with T2DM. Therefore, we examined the vaccination status and anti-SARS-CoV-2 antibody levels to identify the factors that affect the antibody levels in patients with T2DM. This cross-sectional study was conducted at the Dr. Hasan Sadikin Hospital and Bandung Kiwari Hospital, Bandung, West Java, Indonesia, between October and November 2022. Adult participants with and without T2DM were tested for SARS-CoV-2 antibodies using a point-of-care quantitative immunochromatographic assay. We enrolled 289 participants: 201 participants with T2DM and 88 participants without T2DM. The T2DM participants had a lower vaccination rate compared with the non-T2DM participants. However, no significant differences in antibody levels were observed between the two groups. Higher antibody levels among the T2DM participants were associated with mRNA vaccination and a history of COVID-19 illness. The lower antibody response observed among the T2DM participants with chronic obstructive pulmonary disease suggests that such patients may need antibody level measurement and an additional booster vaccine.

## 1. Introduction

COVID-19 has become a global health concern, including in Indonesia. Epidemiologic studies have identified several factors linked with higher mortality in COVID-19, including advanced age, male sex, and pre-existing comorbidities, especially type 2 diabetes mellitus (T2DM) [1]. T2DM patients have a higher risk of contracting COVID-19 and a poor prognosis due to immune response dysregulation, which leads to decreased lymphocyte proliferation, impaired macrophage and neutrophil function, and dysfunction of complement activation [2,3]. Consequently, T2DM patients have lower antibody responses compared to those without T2DM.

Some studies have reported that immunity to COVID-19 lowers the severity of the illness, although this protection wanes in the months after vaccination [4,5]. Recognizing the importance of COVID-19 antibody formation, several studies have evaluated antibody levels post-SARS-CoV-2 vaccination. Previous studies have reported that lower antibody production post-vaccination is associated with diabetes mellitus, especially in patients with poor glycemic control [6,7,8,9]. One study found that the BNT162b2 mRNA vaccine elicits lower neutralizing antibody titers and lower SARS-CoV-2–specific IgG in patients with diabetes compared to non-diabetic patients [8]. Moreover, the CAVEAT study in Italy found that T2DM patients with poor glycemic control showed a significantly reduced neutralizing antibody capacity and worse CD4+ T/cytokine response following COVID-19 vaccination relative to patients with good glycemic control [6]. In addition to diabetes, other factors are associated with a lower immune response, including age, sex, body mass index (BMI), and number of days after vaccination [5,7].

Despite the lower antibody responses in patients with T2DM, few studies have examined post-vaccination antibody responses in T2DM patients and other possible affecting factors. Therefore, this study evaluated antibody levels after vaccination in T2DM patients to identify other possible factors that affect SARS-CoV-2–specific antibody levels.

## 2. Materials and Methods

### 2.1. Study Design and Participants

We conducted this cross-sectional study between October and November 2022 at two hospitals: Hasan Sadikin General Hospital and Bandung Kiwari Hospital, which are the top referral hospital and regional public hospital, respectively, in Bandung, West Java Province, Indonesia. We invited T2DM patients in the outpatient clinic to participate in this study. The inclusion criterion was a diagnosis of T2DM and age ≥18 years, and patients who agreed to participate in the study provided signed informed consent. We used a convenient sampling method to acquire the control group. For every second T2DM patient enrolled, we invited a family member or other accompanying person of similar age (±10% years difference) who did not have T2DM comorbidity to participate in the study. We did not include patients who required hospitalization. Blood was sampled from all enrolled subjects for determination of anti-SARS-CoV-2 antibody levels.

### 2.2. Ethics Approval

This study was approved by the Institutional Review Board (or Ethics Committee) of Hasan Sadikin General Hospital (ethics approval no. LB.02.01/X.6.5/315/2022) and Bandung Kiwari Regional Public Hospital (ethics approval no. PP.01.09/2855-RSUDBK/X/2022).

### 2.3. Data Collection and Outcome

The study obtained clinical information through interviews and medical records. After the participant signed the informed consent, the interviewer used a questionnaire to collect information about COVID-19 vaccination status, type of vaccine, time since the last vaccination, and history of COVID-19. Other research staff extracted baseline demographic data such as age, gender, BMI, and pre-existing comorbidities from the medical records. Comorbidities observed in this study included hypertension, cardiovascular disease, chronic respiratory disease, chronic kidney disease, chronic liver disease, autoimmune disease, and malignancy. In the cases of patients with T2DM, we made additional observations regarding the duration of diabetes, treatments received, and glycemic index (fasting plasma glucose and glycated hemoglobin [HbA1c]).

T2DM was defined as a fasting plasma glucose level > 126 mg/dL (7.0 mmol/L); HbA1c ≥ 6.5; plasma glucose 2 h after an oral glucose tolerance test of ≥200 mg/dL; or a random plasma glucose level of ≥200 mg/dL in a patient with classic symptoms [10]. BMI was classified according to the Asia Pacific criteria: BMI < 18.5 as underweight, 18.5–22.9 as normal, 23–24.9 as overweight, 25–29.9 as pre-obese, and ≥30 as obese [11]. Hypertension was defined as blood pressure ≥140/90 mmHg [12]. Chronic kidney disease was defined according to the Kidney Disease Improving Global Outcomes criteria: decreased glomerular filtration rate of <60 mL/min/1.73 m^2^ for at least 3 months [13]. The glycemic index was defined as controlled if the fasting blood glucose level was between 80 and 130 mg/dL, the blood glucose level 2 h after a meal was <180 mg/dL, or HbA1c was <7% [14]. Vaccination status was divided into categories based on the total number of vaccines already obtained at enrollment: none, 1, 2, or 3. Vaccine type was classified as Sinovac, mRNA ± Sinovac, or AstraZeneca ± Sinovac. Antibody levels were defined as high or low, based on antibody levels ≥4000 BAU/mL or <4000 BAU/mL, respectively.

In this study, we assessed SARS-CoV-2 antibodies using FastBioRBD™, which is produced by Wondfo, Guangzhou Biotech, China, and rebranded for distribution by PT Biofarma Indonesia (Persero). FastBioRBD™ is a fluorescence-based immunochromatographic assay (FIA) that quantifies concentrations of various analytes in human blood or urine. The test result is confirmed as seropositive if it reads ≥1 arbitrary units/mL (AU/mL), with a maximum detection limit of 200 AU/mL. We multiplied the reading result by 20 to acquire a standardized anti-SARS-CoV-2 RBD binding antibody unit (BAU/mL) value. Thus, ≥20 BAU/mL is the cut-off for positive serology, with a maximum detection level for this test of 4000 BAU/mL [15,16].

To validate the FastBioRBD™ antibodies, we tested randomly selected serum samples from 31 participants from both groups using the GenScript c-Pass SARS-CoV-2 neutralization antibody detection kit/sVNT (GenScript c-Pass Biotech, Leiden, The Netherlands) [17]. Validation of FastBioRBD™ compared to the GenScript c-Pass showed a good correlation, with an *r*-value of 0.901 and a 95% confidence interval (CI) of 0.778–0.951 (*p* < 0.0001; Figure S1). A previous study showed that the FIA (Finecare^TM^, Wondfo, Guangzhou, China) is reliable for assessing the antibody response after SARS-CoV-2 vaccination or infection, with an *r*-value of 0.7 (*p* < 0.0001) when validated with sVNT (Genscript c-Pass Biotech) [18].

### 2.4. Statistical Analysis

We described the clinical and demographic characteristics of the enrolled participants using frequency distribution tabulations. SARS-CoV-2 antibody levels were reported as the geometric mean with 95% CI. To compare numeric and categorical variables between groups, we used the Mann–Whitney test and the chi-square test, respectively. We conducted the Kruskal–Wallis test and Dunn’s post hoc test to analyze differences in antibody levels after administration of different vaccine types and doses. Additionally, we used the chi-square test to compare differences in the factors or variables associated with high (≥4000 BAU/mL) versus low (<4000 BAU/mL) antibody levels. These variables were further evaluated by multivariate analysis if they showed an association with a *p*-value <0.25.

We also performed sensitivity analyses to evaluate the factors influencing the antibody levels in the non-T2DM participants. Furthermore, we analyzed the antibody levels in both the T2DM and the non-T2DM patients after the administration of different doses and types of vaccine. All data were analyzed using Statistical Product and Service Solution (SPSS) software, version 25.0, for Windows (IBM Corp.) and GraphPad PRISM, version 9.

## 3. Results

A total of 201 T2DM participants and 88 non-T2DM participants were included in this study. The baseline characteristics of all participants are shown in Table 1. Both groups were predominantly composed of participants younger than 65 years and of the female gender. Co-existing comorbidities, specifically hypertension and cardiovascular disease, were more frequently reported in the T2DM participants compared to the non-T2DM participants (62.2% versus 31.8% and 33.3% versus 21.6%, respectively). No significant difference in BMI was found between the T2DM and non-T2DM groups, with a median (interquartile range [IQR]) of 24.9 (22.6–28.6) and 24.3 (21.9–27.4) for the T2DM and non-T2DM groups, respectively. Subjects in the pre-obese category composed the highest proportion in both the T2DM and non-T2DM groups (33.8% and 33.0%, respectively).

**Figure 1:**
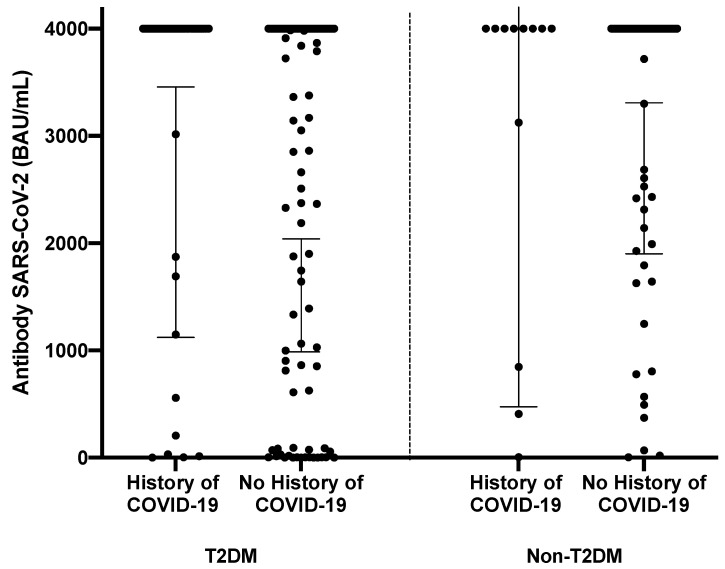
Levels of anti-SARS-CoV-2 RBD antibodies in the T2DM and the non-T2DM participants. (Gray lines denote the geometric mean of each group; thin lines denote the 95% CI of the geometric mean.) There were no significant differences between the T2DM and non-T2DM groups.

Most participants in the two groups had received a COVID-19 vaccination, but the proportion of unvaccinated individuals was slightly higher among the T2DM (30.3%) patients than the non-T2DM patients (19.3%), with a *p*-value of 0.078. The most common vaccine received by the T2DM participants was Sinovac (32.8%), whereas the mRNA ± Sinovac combination was the most common vaccination scheme received by the non-T2DM participants (47.9%). The time since the last vaccination was generally >6 months in both groups. The T2DM group reported a significantly higher history of previous COVID-19 infection compared to the non-T2DM group, at 24.9% and 13.6% (*p* = 0.032), respectively. There was no significant difference in the antibody levels between the T2DM and non-T2DM groups, with or without a history of COVID-19 infection (Table 1, Figure 1). Both groups had similar median values of ≥4000 BAU/mL, with geometric means of 1539 (95% CI 1135–2088) and 2367 (95% CI 1785–3137) for the T2DM and non-T2DM groups, respectively.

In a further analysis, we compared the variables associated with low (<4000 BAU/mL) versus high (≥4000 BAU/mL) anti-SARS-CoV-2 antibody levels in the T2DM participants (Table 2). The factors associated with low levels of anti-SARS-CoV-2 antibodies were vaccination status, type of COVID-19 vaccine, having no history of COVID-19 infection, and having no history of close contact (*p* < 0.05). A multivariate analysis (Table 3) confirmed that having chronic pulmonary disease/COPD was significantly associated with lower antibody levels (odds ratio 4.49; 95% CI 1.49–13.49). Being vaccinated and having a history of COVID-19 infection were inversely correlated with low anti-SARS-CoV-2 antibody levels.

All vaccine types were associated with higher antibody levels. The mRNA ± Sinovac vaccine significantly reduced the risk of having a low anti-SARS-CoV-2 antibody level (<4000 BAU/mL) by 86% (Table 3). A similar analysis of the non-T2DM participants did not reveal any factors associated with lower antibody levels, except being unvaccinated (Supplementary Tables S1 and S2).

We also determined the geometric mean level of the anti-SARS-CoV-2-RBD antibodies and compared the distribution in each category of participants. Patients who were female, had T2DM, and were underweight tended to have lower levels of anti-SARS-CoV-2 antibodies. In the T2DM group, the geometric mean antibody level for female versus male participants was 999.7 (597.1–1674.1) versus 2380.4 (1745.8–3245.6), respectively. Among the T2DM patients with additional comorbidities, those with co-existing chronic pulmonary disease showed the lowest anti-SARS-CoV-2 antibody levels. These differences were not observed among the non-T2DM participants. Co-existing hypertension and cardiovascular comorbidities in the T2DM patients were not significantly associated with lower anti-SARS-CoV-2 antibody levels, with geometric means of 1457.2 (970.3–2188.4) and 1553.9 (887.8–2719.5) for hypertension and cardiovascular disease, respectively. Among all participants, a complete dose of any type of vaccine was associated with significantly higher antibody levels (Supplementary Table S3).

**Figure 2:**
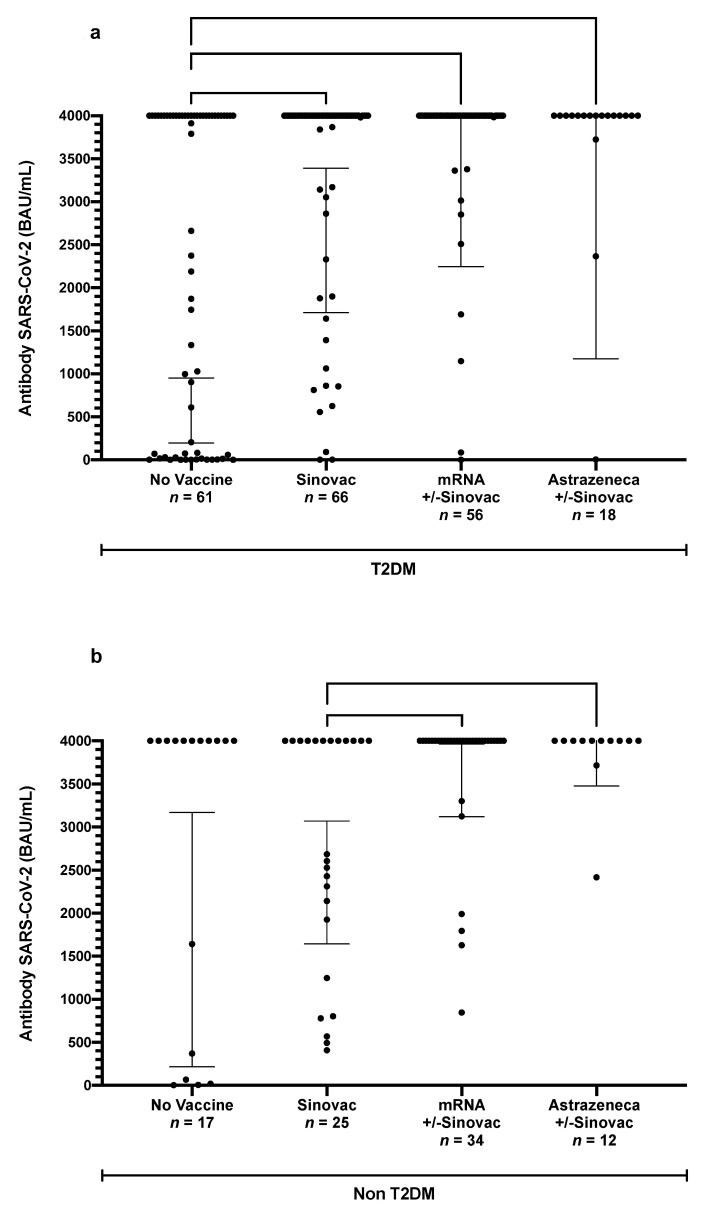
(**a**,**b**) Anti−SARS−CoV−2 antibody levels in the T2DM and the non−T2DM participants based on the type of vaccination. (Gray lines denote the geometric mean for each group; thin lines denote the 95% CI of the geometric mean).

Figure 2 shows the effects of vaccination. Unvaccinated participants exhibited the lowest anti-SARS-CoV-2-RBD antibody levels. A single or complete dose of Sinovac increased the levels of antibodies modestly, whereas a combination scheme that included an mRNA vaccine resulted in the highest antibody levels. In general, we can see a trend that a greater number of vaccine doses received by the participants, regardless of the type, was associated with a higher anti-SARS-CoV-2-RBD level. However, the difference was not always significant, due to the number of samples and the distribution of the antibody levels (Supplementary Figure S2a,b).

A comparison of the different vaccine types revealed that all participants who received a complete dose with a booster showed higher antibody levels compared to those who received no vaccination, regardless of the vaccine type. Specifically, the participants who received the heterologous booster of the mRNA ± Sinovac vaccine type exhibited the highest average antibody levels. Supplementary Figure S3a,b shows the details regarding the individual vaccine types and the administration schemes, including for boosters.

**Figure 3:**
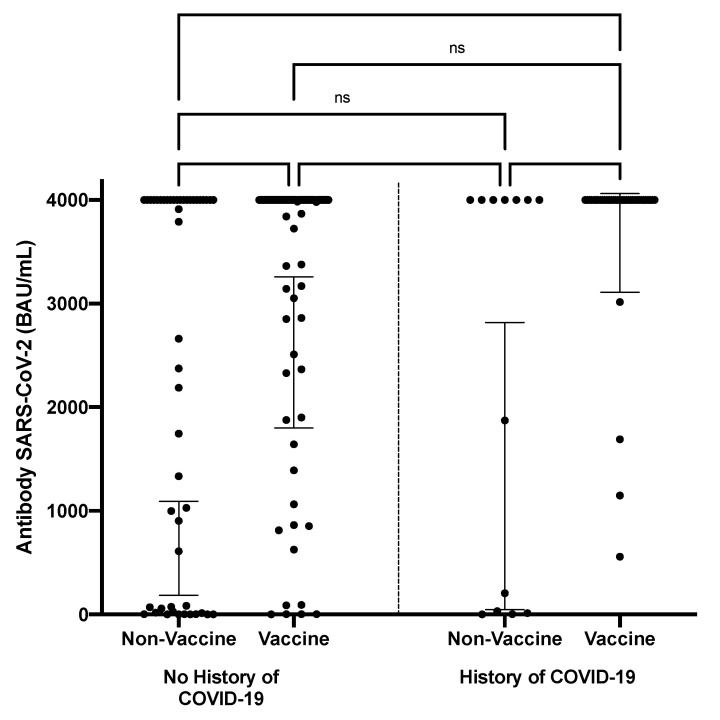
Anti-SARS-CoV-2 antibody levels in T2DM patients based on history of COVID-19 infection (ns = not significant; gray lines denote the geometric mean for each group; thin lines denote the 95% CI of the geometric mean).

Among participants with T2DM, unvaccinated individuals with or without a history of COVID-19 exhibited similar antibody levels. We also observed that the group who had a history of COVID-19 and were vaccinated tended to have higher antibody levels when compared to those without a history of COVID-19. However, this difference was not significant (Figure 3).

## 4. Discussion

The study findings showed a disparity in vaccination rates between participants with and without T2DM comorbidity. The T2DM participants had lower vaccination and booster rates compared to the non-T2DM participants.

The low rate of completion of basic and booster vaccinations in the T2DM patients requires particular attention. We believe that efforts to encourage vaccination among patients with comorbidities in Indonesia were inadequate. In the first months of deployment of the inactivated Sinovac vaccine, there were strict rules that patients with uncontrolled blood glucose (HbA1c ≥7.5%) and hypertension (blood pressure ≥140/90) were not eligible for vaccination [19]. These rules were only softened 8 months after vaccination started, but many T2DM patients still required their attending physician to sign a letter of approval to be vaccinated [19]. Thus, in principal, physicians were generally still concerned about the side effects of the vaccines in patients with comorbidities [20], even though several studies had reported the adequate safety of the COVID-19 vaccines in patients with comorbidities [21,22,23], and another in Greece reported that a more potent vaccine was necessary in people with T2DM because their immune responses are not as robust as those of individuals without T2DM [24].

Tissue damage and impaired cellular functions in T2DM patients induce immune response dysregulation. This led us to hypothesize that participants in the T2DM group would have lower anti-SARS-CoV-2 antibody levels. However, we did not find a significant difference in the anti-SARS-CoV-2 antibody levels between the T2DM and non-T2DM participants. Our study was in concordance with previous studies evaluating humoral immunity in T2DM patients [24,25,26]. A study conducted in Austria (COVAC-DM) also found similar anti-SARS-CoV-2 antibody levels among T2DM patients and healthy controls [25]. However, the CAVEAT study showed that patients with T2DM, especially those with poor glycemic control, had a lower neutralizing antibody capacity [6]. Therefore, we believe our study may not have had sufficient statistical power to identify the differences.

We found high levels of anti-SARS-CoV-2 antibodies among the T2DM and the non-T2DM participants. In our study, 67.8% of the participants had the maximum level of antibodies, despite the lower vaccination completion rate among the T2DM group, contrary to our hypothesis. This could be due to the timing of our study, which was conducted late in the pandemic. High exposure to COVID-19 via natural infection may have also played a role in the development of humoral immunity. This can be explained by data indicating that previous COVID-19 infection in vaccinated individuals induced robust and sustained production of IgG, IgM, IgA, and neutralizing anti-SARS-CoV-2 antibodies [27]. Another study in Kuwait found that vaccinated subjects with a history of COVID-19 infection exhibited higher sustained levels of IgG and neutralizing antibodies, in comparison to a steep decline observed in those without a previous COVID-19 infection [27]. These observations may also explain why we found that the increasing antibody level trend among those who received one, two, or three vaccine doses was not statistically significant.

The T2DM patients who received any type of booster exhibited higher average anti-SARS-CoV-2 antibody levels compared with those who received a full-dose vaccination. In our study, a heterologous booster vaccination induced a higher antibody response. This finding is consistent with previous studies that reported a combination of full doses of the Sinovac vaccine with one dose of the BNT162b2 mRNA vaccine increased the antibody level 104.8-fold and increased the vaccine’s efficacy against severe COVID-19 infections [28,29]. One animal study showed that the administration of one dose, either of the adenovirus vectored vaccine, or the mRNA vaccine after two doses of inactivated vaccine, could induce higher spike-specific T-cell responses and IL-2 expression [30].

Among those with additional pre-existing medical conditions, antibody levels in the T2DM patients were significantly lower among those with co-existing chronic pulmonary disease/COPD. A lower seropositivity rate of both anti-RBD IgG and neutralizing antibodies in COPD patients has been reported [31,32]. The lower immune response is most likely associated with the use of systemic corticosteroids or other immunosuppressants that alter the humoral responses elicited by vaccines [31].

In addition to COPD, participants with T2DM reported a higher frequency of co-existing hypertension and cardiovascular disease. Chronic increased glucose uptake and insulin deficiency make cardiovascular-related disorders, such as hypertension and atherosclerosis, and metabolic disorders, such as obesity and hyperlipidemia, more prevalent in patients with T2DM [33]. In our study, we found hypertension and cardiovascular disease were not associated with lower anti-SARS-CoV-2 antibody levels. Our findings were in concordance with those of a previous study in Italy that reported no significant association between hypertension and antibody levels measured after full-dose vaccination with the BNT162b2 mRNA vaccine [34].

This study has several limitations. As this was a cross-sectional study, the ability to establish causality or determine the temporal relationship between variables was limited compared to a cohort study. Moreover, no matching process was conducted between patients with and without T2DM, which may be a potential confounding factor. However, we attempted to address this limitation by conducting a multivariate analysis with possible confounding factors in patients without T2DM. Additionally, we used a questionnaire to collect data on the participants’ vaccination status (types and times of vaccination); consequently, the possibility of self-reporting bias should also be considered.

## 5. Conclusions

Lower vaccination and booster rates were observed in the participants with T2DM compared with those without T2DM. No significant difference in anti-SARS-CoV-2 antibody levels was observed between the groups. Vaccination, receiving an mRNA-based vaccine, and a history of COVID-19 illness were the most important factors related to high anti-SARS-CoV-2 antibody levels. Lower antibody responses among the T2DM subjects with COPD suggest that these individuals may need antibody level measurement and additional booster vaccines.

## Figures and Tables

**Table 1:** Baseline characteristics of participants.

Variable	T2DM (*n* = 201)	Non-T2DM (*n* = 88)
Age (years), Median (IQR) *	60 (28–84)	56 (21–87)
Age category, *n* (%)		
<65	140 (69.7)	61 (69.3)
≥65	61 (30.3)	27 (30.7)
Gender, *n* (%)		
Female	101 (50.2)	53 (60.2)
Male	100 (49.8)	35 (39.8)
Comorbidity, *n* (%)		
Hypertension **	125 (62.2)	28 (31.8)
Obesity	25 (12.4)	5 (5.7)
Cardiovascular disease *	67 (33.3)	19 (21.6)
Chronic pulmonary disease	19 (9.5)	3 (3.4)
Chronic kidney disease	9 (4.5)	1 (1.1)
Chronic liver disease	10 (5.0)	3 (3.4)
Autoimmune disease	4 (2.0)	2 (2.3)
Malignancy	17 (8.5)	2 (2.3)
Body mass index, *n* (%)		
Underweight	9 (4.5)	7 (8.0)
Normal	48 (23.9)	24 (27.3)
Overweight	44 (21.9)	19 (21.6)
Pre-obese	68 (33.8)	29 (33.0)
Obese	32 (15.9)	9 (10.2)
Vaccination status, *n* (%)		
Never	61 (30.3)	17 (19.3)
1 time	14 (7.0)	3 (3.4)
2 times	60 (29.9)	28 (31.8)
3 times	66 (32.8)	40 (45.5)
Type of COVID-19 vaccine, *n* (%) **		
None	61 (30.3)	17 (19.3)
Sinovac	66 (32.8)	25 (35.2)
mRNA ± Sinovac	56 (27.9)	34 (47.9)
AstraZeneca ± Sinovac	18 (9.0)	12 (16.9)
Time since last vaccination (months), Median (IQR)	11 (0–30)	9 (2–19)
Time since last vaccination category (months), *n* (%)		
<3	12 (8.6)	3 (4.2)
3–6	24 (17.1)	18 (25.4)
>6	104 (74.3)	50 (70.4)
History of COVID-19 infection, *n* (%) *		
Yes	50 (24.9)	12 (13.6)
No	151 (75.1)	76 (86.4)
History of close contact, *n* (%)		
Yes	48 (23.9)	13 (14.8)
No	153 (76.1)	75 (85.2)
Antibody level (BAU/mL), geometric mean (95% CI)	1539 (1135–2088)	2367 (1785–3137)
Antibody level category (BAU/mL), *n* (%)		
<4000	66 (32.8)	27 (30.7)
≥4000	135 (67.2)	61 (69.3)

T2DM = type 2 diabetes mellitus; * *p* < 0.05; ** *p* < 0.001.

**Table 2:** Bivariate analysis of the factors related to high anti-SARS-CoV-2 antibody levels among participants with T2DM.

Variable	SARS-CoV-2 Antibody Level (BAU/mL)		*p*-Value
	<4000 (*n* = 66)	≥4000 (*n* = 135)	
Age (years), Median (IQR)	60 (51–67)	60 (54–65)	0.742
Age category, *n* (%)			
<65	44 (66.7)	96 (71.1)	0.520
≥65	22 (33.3)	39 (28.9)	
Gender, *n* (%)			
Female	38 (57.6)	63 (46.7)	0.146
Male	28 (42.4)	72 (53.3)	
Comorbidity, *n* (%)			
Hypertension			
Yes	42 (63.6)	83 (61.5)	0.767
No	24 (36.4)	52 (38.5)	
Obesity			
Yes	8 (12.1)	17 (12.6)	0.924
No	58 (87.9)	118 (87.4)	
Cardiovascular disease			
Yes	20 (30.3)	47 (34.8)	0.524
No	46 (69.7)	88 (65.2)	
Chronic pulmonary disease			
Yes	10 (15.1)	9 (6.7)	0.053
No	56 (64.8)	126 (93.3)	
Chronic kidney disease			
Yes	3 (4.5)	6 (4.4)	1.000
No	63 (95.5)	129 (95.6)	
Chronic liver disease			
Yes	3 (4.5)	7 (5.2)	1.000
No	63 (95.5)	128 (94.8)	
Autoimmune disease			
Yes	3 (4.5)	1 (0.7)	0.105
No	63 (95.5)	134 (99.3)	
Malignancy			
Yes	5 (3.9)	12 (8.9)	0.753
No	123 (96.1)	123 (91.1)	
Body mass index, *n* (%)			
Underweight	4 (6.1)	5 (3.7)	0.802
Normal	18 (27.3)	30 (22.2)	
Overweight	13 (19.7)	31 (22.9)	
Pre-obese	20 (30.3)	48 (35.6)	
Obese	11 (16.6)	21 (15.6)	
Duration of T2DM, years, *n* (%)			
<5	33 (51.6)	72 (53.7)	0.775
≥5	31 (48.4)	62 (46.3)	
T2DM therapy, *n* (%)			
Oral anti-hyperglycemic	27 (40.9)	57 (42.2)	0.318
Injection anti-hyperglycemic	24 (36.4)	48 (35.6)	
Combination	11 (16.7)	28 (20.7)	
Without therapy	4 (6.0)	2 (1.5)	
Glycemic index, *n* (%)			
Controlled	7 (10.9)	23 (17.3)	0.245
Uncontrolled	57 (89.1)	110 (82.7)	
Vaccination status, *n* (%)			
Never	32 (48.5)	29 (21.5)	<0.001 **
1 time	4 (6.1)	10 (7.4)	
2 times	19 (28.8)	41 (30.4)	
3 times	11 (16.6)	55 (40.7)	
Type of COVID-19 vaccine, *n* (%)			<0.001 **
None	32 (48.5)	29 (21.5)	
Sinovac	21 (31.8)	45 (33.3)	
mRNA ± Sinovac	10 (15.2)	46 (34.1)	
AstraZeneca ± Sinovac	3 (4.5)	15 (11.1)	
Time since last vaccination (months), Median (IQR)	11 (7–14)	10 (6–13)	0.294
Time since last vaccination category (months), *n* (%)			0.697
<3	3 (8.8)	12 (11.3)	
3–6	5 (14.7)	21 (19.8)	
>6	26 (76.5)	73 (68.9)	
History of COVID-19 infection, *n* (%)			0.026 *
Yes	10 (15.2)	40 (29.6)	
No	56 (84.8)	95 (70.4)	
History of close contact, *n* (%)			0.001 *
Yes	6 (9.1)	42 (31.1)	
No	60 (90.9)	93 (68.9)	

T2DM = type 2 diabetes mellitus; * *p* < 0.05; ** *p* < 0.001.

**Table 3:** Bivariate and multivariate analysis of the factors associated with low anti-SARS-CoV-2 antibody levels (<4000 BAU/mL) among participants with T2DM.

Variable	Bivariate		Multivariate	
	Odds Ratio	*p*	Adjusted Odds Ratio	*p*
Gender (female)	1.55 (0.86–2.81)	0.147	1.28 (0.66–2.49)	0.464
Comorbidity				
Chronic pulmonary disease	2.50 (0.96–6.49)	0.060	4.49 (1.49–13.49)	0.007 *
Autoimmune disease	6.38 (0.65–62.57)	0.112	10.34 (0.97–109.77)	0.053
Glycemic index (uncontrolled)	1.69 (0.68–4.17)	0.257	1.88 (0.70–5.03)	0.210
Type of COVID-19 vaccine				
Never	Ref		Ref	
Sinovac	0.42 (0.21–0.87)	0.019 *	0.39 (0.18–0.83)	0.015 *
mRNA ± Sinovac	0.20 (0.08–0.46)	<0.001 *	0.14 (0.06–0.81)	<0.001 *
AstraZeneca ± Sinovac	0.18 (0.05–0.69)	0.012 *	0.21 (0.05–0.81)	0.023 *
History of COVID-19 infection	0.42 (0.20–0.91)	0.029 *	0.43 (0.19–0.99)	0.049 *

Dependent variable: antibody level anti-SARS-CoV-2 < 4000 BAU/mL, * Statistically significant.

## Data Availability

The data used to support the findings of this study were included in the article.

## Supplementary Materials

The following supporting information can be downloaded at: https://www.mdpi.com/article/10.3390/vaccines11091424/s1, Figure S1: Linear correlation of FastBioRBD^tm^ compared to the surrogate viral neutralization test (SVNT) in 31 randomly selected samples; Figure S2. a & b. Anti-SARS-CoV-2-RBD antibody levels in the T2DM and the non-T2DM par-ticipants based on vaccination status. (ns = non-significant; * *p* < 0.05; ** *p* < 0.001; gray lines de-note the geometric mean for each group; thin lines denote the 95% CI of the geometric mean); Figure S3. (a). Levels of anti-SARS-CoV-2 antibody by dose and type of vaccine in the T2DM group. (Gray lines denote the geometric mean [95% CI] with * *p* < 0.05; ** *p* < 0.001) (b). Levels of anti-SARS-CoV-2 antibody by dose and type of vaccine in the non-T2DM group. (Gray lines de-note the geometric mean [95% CI] with * *p* < 0.05). Table S1: Bivariate analysis of influencing factors associated with low (<4000 BAU/mL) anti-SARS-CoV-2 antibody levels in the non-T2DM participants; Table S2. Bivariate and multivariate analysis of the influencing factors associated with low anti-SARS-CoV-2 antibody levels (<4000 BAU/mL) in the non-T2DM participants; Table S3. Anti-SARS-CoV-2 antibody levels in the T2DM and the non-T2DM participants.

## References

[1] Dessie Z.G., Zewotir T. (2021). Mortality-related risk factors of COVID-19: A systematic review and meta-analysis of 42 studies and 423,117 patients. BMC Infect. Dis..

[2] Gold M.S., Sehayek D., Gabrielli S., Zhang X., McCusker C., Ben-Shoshan M. (2020). COVID-19 and comorbidities: A systematic review and meta-analysis. Postgrad. Med..

[3] Yamamoto S., Saito M., Nagai E., Toriuchi K., Nagai H., Yotsuyanagi H., Nakagama Y., Kido Y., Adachi E. (2020). Antibody response to SARS-CoV-2 in people living with HIV. J. Microbiol. Immunol. Infect. Wei Mian Yu Gan Ran Za Zhi.

[4] Ng O., Marimuthu K., Lim N., Lim Z., Thevasagayam N., Koh V., Chiew C., Ma S., Koh M., Low P. (2022). Analysis of COVID-19 Incidence and Severity among Adults Vaccinated with 2-Dose mRNA COVID-19 or Inactivated SARS-CoV-2 Vaccines with and without Boosters in Singapore. JAMA Netw. Open.

[5] Ssentongo P., Ssentongo A.E., Voleti N., Groff D., Sun A., Ba D.M., Nunez J., Parent L.J., Chinchilli V.M., Paules C.I. (2022). SARS-CoV-2 vaccine effectiveness against infection, symptomatic and severe COVID-19: A systematic review and meta-analysis. BMC Infect. Dis..

[6] Marfella R., D’Onofrio N., Sardu C., Scisciola L., Maggi P., Coppola N., Romano C., Messina V., Turriziani F., Siniscalchi M. (2022). Does poor glycaemic control affect the immunogenicity of the COVID-19 vaccination in patients with type 2 diabetes: The CAVEAT study. Diabetes Obes. Metab..

[7] Xiang F., Long B., He J., Cheng F., Zhang S., Liu Q., Chen Z., Li H., Chen M., Peng M. (2023). Impaired antibody responses were observed in patients with type 2 diabetes mellitus after receiving the inactivated COVID-19 vaccines. Virol. J..

[8] Ali H., Alterki A., Sindhu S., Alahmad B., Hammad M., Al-Sabah S., Alghounaim M., Jamal M.H., Aldei A., Mairza M.J. (2021). Robust antibody levels in both diabetic and non-diabetic individuals after BNT162b2 mRNA COVID-19 vaccination. Front. Immunol..

[9] Soetedjo N.N.M., Iryaningrum M.R., Lawrensia S., Permana H. (2022). Antibody response following SARS-CoV-2 vaccination among patients with type 2 diabetes mellitus: A systematic review. Diabetes Metab. Syndr. Clin. Res. Rev..

[10] American Diabetes Association (2009). Diagnosis and classification of diabetes mellitus. Diabetes Care.

[11] World Health Organization (2000). The Asia-Pacific Perspective: Redefining Obesity and Its Treatment.

[12] Whelton P.K., Williams B. (2018). The 2018 European society of cardiology/European society of hypertension and 2017 American college of cardiology/American heart association blood pressure guidelines: More similar than different. JAMA.

[13] Levey A.S., Eckardt K.-U., Tsukamoto Y., Levin A., Coresh J., Rossert J., Zeeuw D.D., Hostetter T.H., Lameire N., Eknoyan G. (2005). Definition and classification of chronic kidney disease: A position statement from Kidney Disease: Improving Global Outcomes (KDIGO). Kidney Int..

[14] American Diabetes Association Professional Practice Committee (2022). 6. Glycemic targets: Standards of medical care in diabetes—2022. Diabetes Care.

[15] Guangzhou Wondfo Biotech Co. (China) (2019). Result Report on Finecare 2019-nCoV RBD Antibody Test.

[16] Infantino M., Pieri M., Nuccetelli M., Grossi V., Lari B., Tomassetti F., Calugi G., Pancani S., Benucci M., Casprini P. (2021). The WHO International Standard for COVID-19 serological tests: Towards harmonization of anti-spike assays. Int. Immunopharmacol..

[17] GenScript (2022). cPass SARS-CoV-2 Neutralization Antibody Detection Kit Instruction for Use.

[18] Shurrab F.M., Younes N., Al-Sadeq D.W., Liu N., Qotba H., Abu-Raddad L.J., Nasrallah G.K. (2022). Performance evaluation of novel fluorescent-based lateral flow immunoassay (LFIA) for rapid detection and quantification of total anti-SARS-CoV-2 S-RBD binding antibodies in infected individuals. Int. J. Infect. Dis..

[19] Kementerian Kesehatan Republik Indonesia (2021). Petunjuk Teknis Pelaksanaan Vaksinasi dalam Rangka Penanggulangan Pandemi Corona Virus Disease 2019 (COVID-19).

[20] Biswas N., Mustapha T., Khubchandani J., Price J.H. (2021). The nature and extent of COVID-19 vaccination hesitancy in healthcare workers. J. Community Health.

[21] Frater J., Ewer K.J., Ogbe A., Pace M., Adele S., Adland E., Alagaratnam J., Aley P.K., Ali M., Ansari M.A. (2021). Safety and immunogenicity of the ChAdOx1 nCoV-19 (AZD1222) vaccine against SARS-CoV-2 in HIV infection: A single-arm substudy of a phase 2/3 clinical trial. Lancet HIV.

[22] Choi W.S., Cheong H.J. (2021). COVID-19 vaccination for people with comorbidities. Infect. Chemother..

[23] Yan Z., Yang M., Lai C.-L. (2021). COVID-19 vaccinations: A comprehensive review of their safety and efficacy in special populations. Vaccines.

[24] Papadokostaki E., Tentolouris A., Anastasiou I.A., Psichogiou M., Iliaki E., Eleftheriadou I., Hatzakis A., Tentolouris N. (2022). Immunogenicity of SARS-CoV-2 BNT162b2 vaccine in people with diabetes: A prospective observational study. Vaccines.

[25] Sourij C., Tripolt N.J., Aziz F., Aberer F., Forstner P., Obermayer A.M., Kojzar H., Kleinhappl B., Pferschy P.N., Mader J.K. (2022). Humoral immune response to COVID-19 vaccination in diabetes is age-dependent but independent of type of diabetes and glycaemic control: The prospective COVAC-DM cohort study. Diabetes Obes. Metab..

[26] Otsubo N., Fukuda T., Beppu H., Maki C., Yasui F., Hanawa T., Sugita C., Murakami M., Yamada T., Kohara M. (2023). Reduced antibody response to SARS-CoV-2 in COVID-19 patients with newly diagnosed diabetes: A retrospective observational study. BMC Endocr. Disord..

[27] Ali H., Alahmad B., Al-Shammari A.A., Alterki A., Hammad M., Cherian P., Alkhairi I., Sindhu S., Thanaraj T.A., Mohammad A. (2021). Previous COVID-19 infection and antibody levels after vaccination. Front. Public Health.

[28] Cerqueira-Silva T., Katikireddi S.V., de Araujo Oliveira V., Flores-Ortiz R., Júnior J.B., Paixão E.S., Robertson C., Penna G.O., Werneck G.L., Barreto M.L. (2022). Vaccine effectiveness of heterologous CoronaVac plus BNT162b2 in Brazil. Nat. Med..

[29] Çağlayan D., Süner A.F., Şiyve N., Güzel I., Irmak Ç., Işik E., Appak Ö., Çelik M., Öztürk G., Alp Çavuş S. (2022). An analysis of antibody response following the second dose of CoronaVac and humoral response after booster dose with BNT162b2 or CoronaVac among healthcare workers in Turkey. J. Med. Virol..

[30] Zhang J., He Q., An C., Mao Q., Gao F., Bian L., Wu X., Wang Q., Liu P., Song L. (2021). Boosting with heterologous vaccines effectively improves protective immune responses of the inactivated SARS-CoV-2 vaccine. Emerg. Microbes Infect..

[31] Harboe Z.B., Hamm S.R., Pérez-Alós L., Sivapalan P., Priemé H., Wilcke T., Kjeldgaard P., Shaker S., Jordan A.S., Møller D.L. (2022). Antibody responses and risk factors associated with impaired immunological outcomes following two doses of BNT162b2 COVID-19 vaccination in patients with chronic pulmonary diseases. BMJ Open Respir. Res..

[32] Yang L., Xu L., Guo Q., Deng B., Hong Y., Wang L., Wang Y., Jiang D., Ren H. (2023). Immune responses to inactivated COVID-19 vaccine were decreased in Chinese patients with chronic respiratory diseases. Int. J. Med. Sci..

[33] Chen Y., Zhao X., Wu H. (2019). Metabolic stress and cardiovascular disease in diabetes mellitus: The role of protein O-GlcNAc modification. Arterioscler. Thromb. Vasc. Biol..

[34] Pellini R., Venuti A., Pimpinelli F., Abril E., Blandino G., Campo F., Conti L., De Virgilio A., De Marco F., Di Domenico E.G. (2021). Initial observations on age, gender, BMI and hypertension in antibody responses to SARS-CoV-2 BNT162b2 vaccine. EClinicalMedicine.

