# Supplementary material for: Anti-SARS-CoV-2 Antibody Level Is Associated with a History of COVID-19 Infection and mRNA Vaccination in Patients with Diabetes
Source: Vaccines (Basel). 2023 Aug 27;11(9):1424. doi: 10.3390/vaccines11091424 (PMC10536458; doi:10.3390/vaccines11091424)
Supplement: Supplementary file 1 [file vaccines-11-01424-s001.zip › vaccines-2520718-supplementary.pdf]

Table S1. Bivariate analysis of influencing factors associated with low (< 4000 BAU/mL) anti SARS-CoV-2 antibody level of non-T2DM participants.

Variables	SARS-CoV-2 Antibody Level (BAU/mL)		p-value
	**<4000** (n=27)	**≥4000** (n=61)	
**Age (years), Median (IQR)**	57 (45 – 68)	56 (41 – 67)	0.480
**Age category, n (%)**			0.390
<65	17 (63.0)	44 (72.1)	
≥65	10 (37.0)	17 (27.9)	
**Gender, n (%)**			0.727
Female	17 (63.0)	36 (59.0)	
Male	10 (37.0)	25 (41.0)	
**Comorbidity, n (%)**			
Hypertension			0.091
Yes	12 (44.4)	16 (26.2)	
No	15 (55.6)	45 (73.8)	
Obesity			0.640
Yes	2 (7.4)	3 (4.9)	
No	25 (92.6)	58 (95.1)	
Cardiovascular disease			0.924
Yes	6 (22.2)	13 (21.3)	
No	21 (77.8)	48 (78.7)	
Chronic pulmonary disease			1.000
Yes	1 (3.7)	2 (3.3)	
No	26 (96.3)	59 (96.7)	
Chronic kidney disease			0.307
Yes	1 (3.7)	0 (0)	
No	26 (96.3)	61 (100)	
Chronic liver disease			0.222
Yes	2 (7.4)	1 (1.6)	
No	25 (92.6)	60 (98.4)	
Autoimmune disease			1.000
Yes	0 (0)	2 (3.3)	
No	27 (100)	59 (96.7)	
Malignancy			0.522
Yes	1 (3.7)	1 (1.6)	
No	26 (96.3)	60 (98.4)	
**Body mass index, n (%)**			0.735
Underweight	1 (3.7)	6 (9.8)	
Normal	6 (22.2)	18 (29.5)	
Overweight	6 (22.2)	13 (21.3)	
Pre-obese	11 (40.7)	18 (29.5)	
Obese	3 (11.1)	6 (9.8)	
**Vaccination status, n (%)**			0.163

Never	6 (22.2)	11 (18.0)	
1 time	2 (7.4)	1 (1.6)	
2 times	11 (40.7)	17 (27.9)	
3 times	8 (29.6)	32 (52.5)	
**Type of COVID-19 vaccine, n (%)**			**0.025***
Never	6 (22.2)	11 (18.0)	
Sinovac	13 (48.1)	12 (19.7)	
mRNA ± sinovac	6 (22.2)	28 (45.9)	
Astra zeneca ± sinovac	2 (7.4)	10 (16.4)	
**Duration from the last vaccination (months), Median (IQR)**	13 (9 – 15)	8 (6 – 13)	**0.040***
**Duration from the last vaccination category (months), n (%)**			0.138
< 3	1 (4.8)	2 (4.0)	
3-6	2 (9.5)	16 (32.0)	
> 6	18 (85.7)	32 (64.0)	
**History of COVID-19 infection, n (%)**			1.000
Yes	4 (14.8)	8 (13.1)	
No	23 (85.2)	53 (86.9)	
**History of close contact, n (%)**			0.329
Yes	2 (7.4)	11 (18.0)	
No	25 (92.6)	50 (82.0)	

Table S2. Bivariate and multivariate analysis of influencing factors associated with low anti SARS-CoV-2 antibody level (<4000 BAU/mL) of participants with non-T2DM

Variables	Bivariate	
	**Odds Ratio**	**p**
**Comorbidity**		
Hypertension	2.25 (0.87 – 5.82)	0.094
Chronic liver disease	4.80 (0.42 – 55.37)	0.209
**Type of COVID-19 vaccine**		
Never	Ref	
Sinovac	1.99 (0.56 – 7.05)	0.288
mRNA ± Sinovac	0.39 (0.10 – 1.48)	0.168
Aztrazeneca ± Sinovac	0.37 (0.06 – 2.25)	0.279

Dependent variable: antibody level SARS-CoV-2 <4000 BAU/ml

Table S3. Anti SARS-CoV-2 antibody level of T2DM and non-T2DM participants

Variables	n	**T2DM (*n* = 201) Anti SARS-CoV-2 Ab** BAU/mL	n	**Non-T2DM (*n* = 88) Anti-SARS-CoV-2 Ab** BAU/mL	**T2DM vs Non-T2DM (p-value)**
**Age category**					
<65	140	1682.9 (1199.3– 2361.6)	61	2478.5 (1759.5 – 3491.2)	0.477
≥65	61	1254.5 (654.9 – 2402.9)	27	2132.7 (1258.9 – 3612.9)	0.937
**Gender**					
Female	101	999.7 (597.1 – 1674.1)	53	2105.7 (1344.9 – 3296.8)	0.193
Male	100	2380.4 (1745.8–3245.6) *	35	2825.1 (2255.9 – 3538)	0.646
**Body mass index**					
Underweight	9	521.9 (56.7 – 4806.2)	7	3746.3 (3191.3 – 4397.8)	0.085
Normal	48	1673.4 (881.4 – 3177.2)	24	2545.1 (1426.6 – 4540.5)	0.420
Overweight	44	2248.8 (1371.4 –3687.5)	19	2635.3 (1634.7 – 4248.2)	0.818
Pre-obese	68	1432.8 (833.1 – 2464.1)	29	2132.7 (1351 – 3366.7)	0.834
Obese	32	1273.3 (513.2 – 3159.1)	9	1521.2 (228.5 – 10127)	0.960
**Duration from the last vaccination category (months)**					
< 3	12	2732.6 (1352.9 –5519.3)	3	3135.3 (1099.3 – 8942)	0.814
3-6	24	2660.7 (1366.4 –5181.3)	18	3602.7 (2972.5 – 4366.5)	0.552
> 6	104	2679.4 (2080.9 –3449.9)	50	2859.9 (2409 – 3395.1)	0.225
**History of COVID-19 infection**					
Yes	50	1969.1 (1121.7– 3456.7)	12	1641 (475.4 – 5664.7)	0.454
No	187	1622.8 (1186.6– 2219.2)	76	2507.7 (1900.8 – 3308.4)	0.261
**History of close contact**					
Yes	48	2627.9 (1568.7– 4402.1) *	13	2018.3 (644.7 – 6318.5)	0.436
No	153	1301.6 (903.3 – 1875.5)	75	2433.1 (1836.1 – 3224.1)	0.221
**Comorbidity**					
Hypertension	125	1457.2 (970.3 – 2188.4)	28	2024.7 (1217.9 – 3365.8)	0.556
Cardiovascular disease	67	1553.9 (887.8 – 2719.5)	19	1849.2 (872.5 – 3919.3)	0.609
Chronic respiratory disease	19	1449.6 (865.5 – 2427.9)	3	1809.7 (59.6 – 54916)	0.810
Chronic liver disease	10	3340.5 (2610 – 4275.5)	3	2650.9 (1047 – 6711.9)	0.275
Malignancy	17	2974.1 (2211.3 – 4000)	2	3109.9 (126.9 – 76169)	0.894
Obesity	25	1617.1 (635.5 – 4115.1)	9	1521.2 (228.5 – 10127)	0.793
**Vaccination status among subjects**					
**No vaccination**	61	430,95 (195,06 – 952,10)	17	693,70 (145,37 – 3310,4)	0.410
**Single dose Sinovac**	13	2027,9 (582,15 – 7063,9)	3	1357,7 (132,78 – 13882)	0.097
**Full-dose vaccination without booster**					
Sinovac	50	2456,6 (1735,5 – 3477,3) *	20	1694,9 (908,86 – 3160,7)	0.056

mRNA	9	3780,7 (3448,6 – 4144,8) *	6	4000,0 (4000,0 – 4000,0) *	0.269
**Full-dose vaccination with booster**					
Sinovac + sinovac	3	3691,0 (2611,7 – 5216,4)	2	4000,0 (4000,0 –4000,0) *	0.495
Sinovac + mRNA	38	3015,0 (1991,0 – 4565,8) *	25	1635,2 (906,21– 2950,5)	0.008*
Sinovac + Astra zeneca	15	3981,0 (3940,4 – 4021,9) *	10	2074,0 (852,71 – 5044,4)	0.031*
mRNA + mRNA	7	4000,0 (4000,0–4000,0) *	3	1473,6 (20,064 – 108229)	0.133

Data is presented as geometric mean (95% CI). *: significant difference compared to the lowest value in each category group.

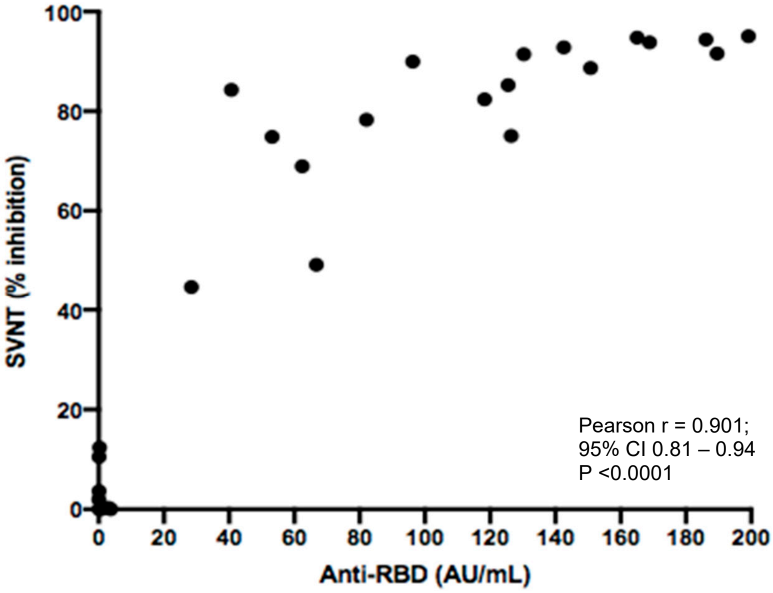

Figure S1. Linear correlation of anti-RBD FastBioRBD[tm] compared to the Surrogate Viral Neutralization Test (SVNT) in 31 randomly selected samples.

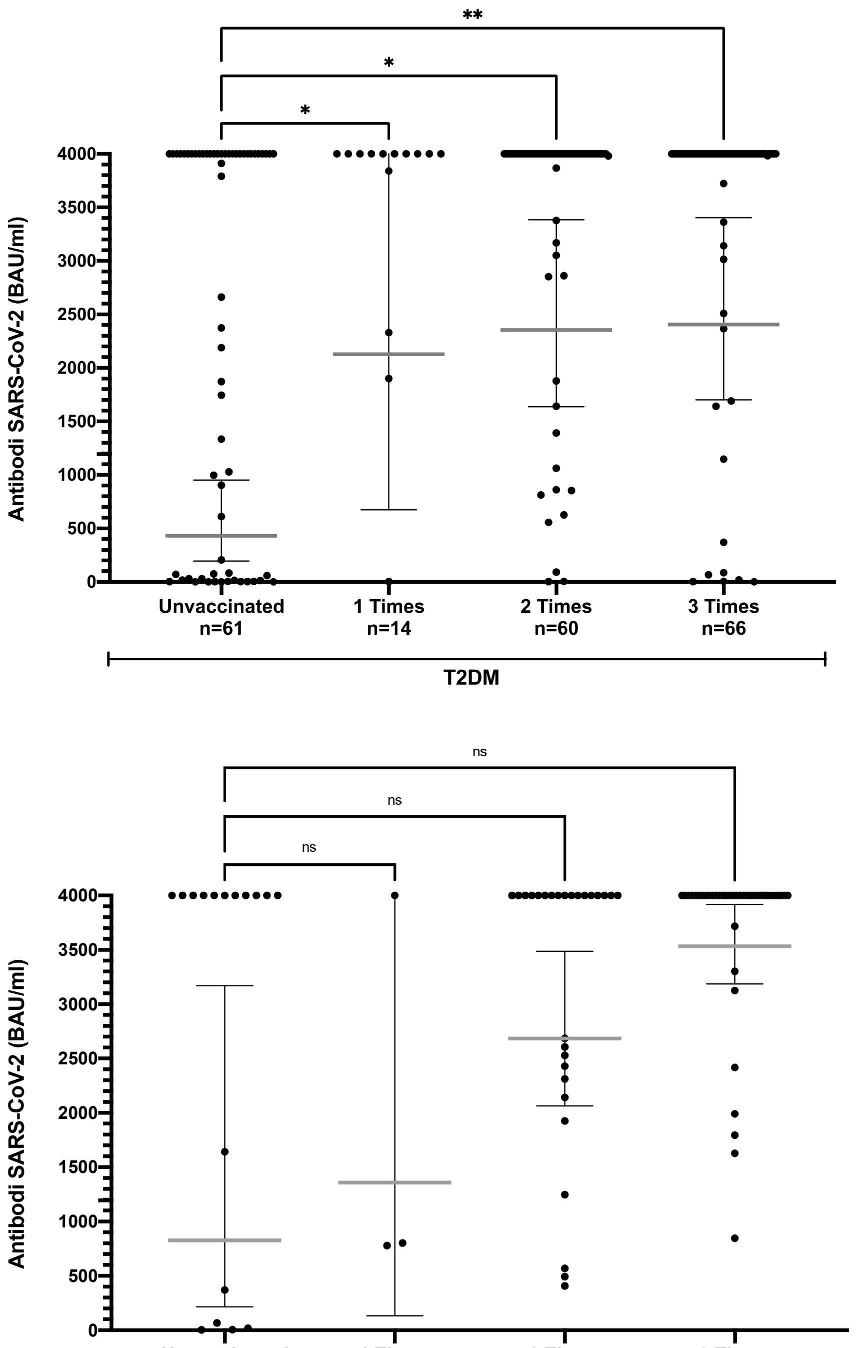

Figure S2a & b. Anti-SARS-CoV-2-RBD Antibody Level in T2DM and non T2DM participants based on vaccination status. (ns=non-significant; *= p<0.05; **= p<0.001; grey lines depicted the geometric mean of each group; thin lines depicted the 95% CI of the geometric mean)

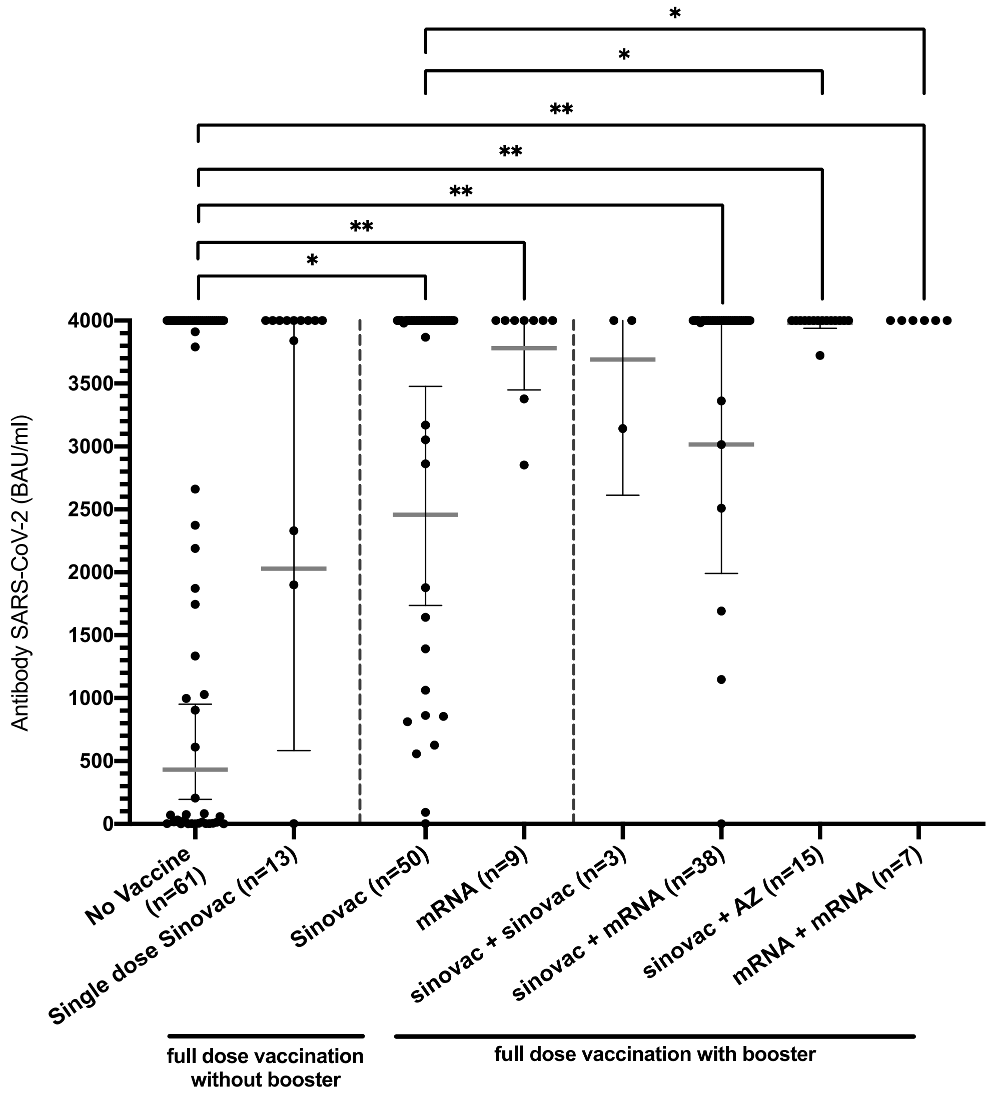

Figure S3a. Level of anti-SARS-CoV-2 antibody between different doses and types of vaccination in T2DM Group. (Grey lines presented the geometric mean (95% CI) with *: p<0.05; **: p<0.001)

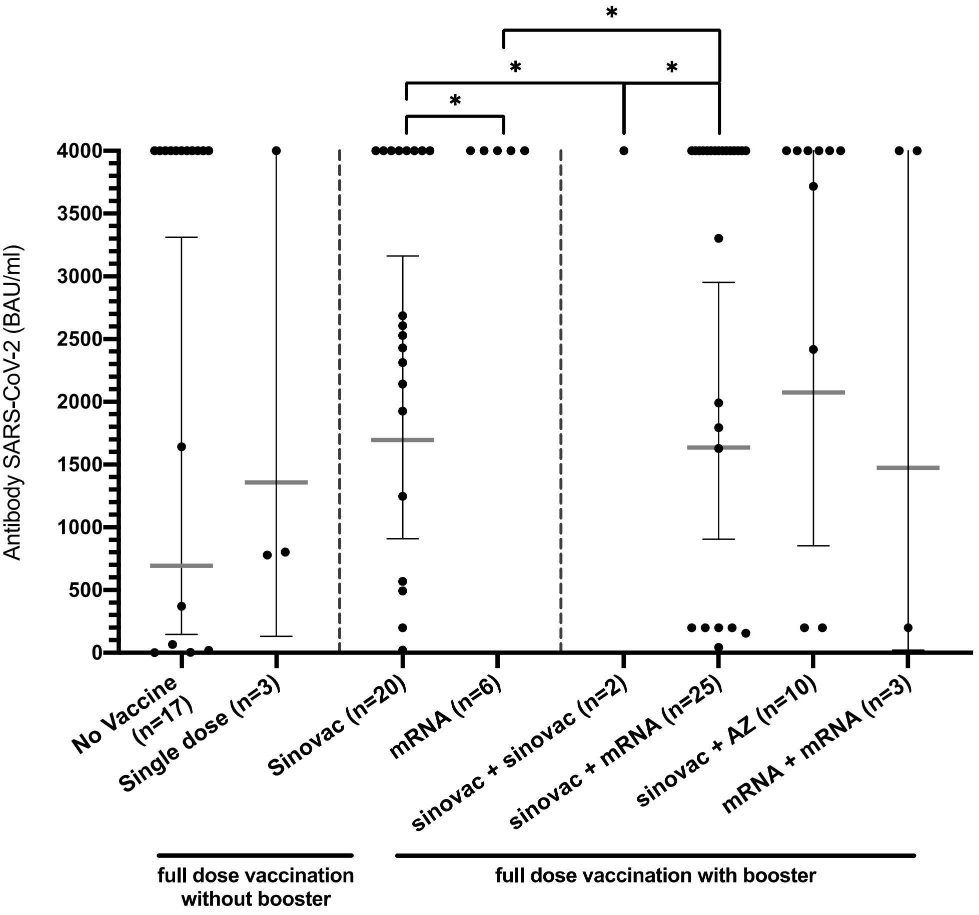

FigureS3b. Level of anti-SARS-CoV-2 antibody between different doses and types of vaccination in non-T2DM Group. (Grey lines presented the geometric mean (95% CI) with *: $p<0.05$)
